# Atypical Cryptococcal Pneumonia in an Immunocompetent Host: A Case Report and Review of Diagnostic and Therapeutic Challenges

**DOI:** 10.7759/cureus.81600

**Published:** 2025-04-02

**Authors:** Gaurav Bector, Mahyar Toofantabrizi, Chetan Kapila, Kushal Sood, Prateek Jain, Tejal Mehta, Gaurika Joshi, Akashdeep Singh Khehra, Fnu Kalpana

**Affiliations:** 1 Medicine and Surgery, Dayanand Medical College and Hospital, Ludhiana, IND; 2 Internal Medicine, MedStar Union Memorial Hospital, Baltimore, USA; 3 Emergency, Fortis Hospital, Ludhiana, IND; 4 Internal Medicine, Fortis Hospital, Ludhiana, IND; 5 Internal Medicine, All India Institute of Medical Sciences, Rishikesh, IND; 6 Medicine and Surgery, Maharishi Markandeshwar Institute of Medical Sciences (MMIMSR), Ludhiana, IND; 7 Internal Medicine, Dayanand Medical College and Hospital, Ludhiana, IND; 8 Internal Medicine, Pandit Bhagwat Dayal Sharma Post Graduate Institute of Medical Sciences, Rohtak, IND

**Keywords:** cryptococcal pneumonia, cryptococcus gattii, fungal infection, immunocompetent host, pulmonary fungal infections

## Abstract

Cryptococcal infections, commonly associated with immunocompromised hosts, are rare but increasingly recognized in immunocompetent individuals. This case report describes a 39-year-old immunocompetent male presenting with cryptococcal pneumonia, initially misdiagnosed as bacterial pneumonia. The patient's persistent symptoms and diagnostic complexities highlight the importance of including cryptococcal infection in the differential diagnosis of chronic respiratory conditions unresponsive to conventional treatment. Treatment with fluconazole was effective, underscoring its role in managing isolated pulmonary cryptococcosis. This report aims to increase clinician awareness of the atypical presentations of cryptococcal infections in healthy hosts and the necessity of thorough diagnostic protocols for appropriate management.

## Introduction

Cryptococcosis, primarily caused by the encapsulated fungi *Cryptococcus neoformans* and *Cryptococcus gattii*, is a life-threatening fungal infection that traditionally affects immunocompromised individuals, particularly those with HIV/AIDS, transplant recipients, or patients receiving prolonged corticosteroid therapy [1]. These fungi found ubiquitously in the environment are transmitted via inhalation of airborne spores, with infections most frequently localizing within the pulmonary system before potentially disseminating to the central nervous system (CNS). Disseminated cryptococcosis, especially cryptococcal meningoencephalitis, carries a high mortality rate, underscoring the clinical importance of early and accurate diagnosis [2,3].

Cryptococcal infection is generally rare among immunocompetent individuals, where the immune system is typically able to contain the pathogen, preventing systemic spread. Nevertheless, recent studies have begun documenting cases of primary pulmonary cryptococcosis in immunocompetent hosts, often leading to diagnostic challenges due to its atypical presentation in this patient population [4]. 

Epidemiology

The global incidence of cryptococcosis closely aligns with the prevalence of HIV/AIDS. Cryptococcal meningitis, in particular, remains a significant cause of mortality among individuals with untreated HIV, with the World Health Organization estimating over 220,000 cases annually, leading to approximately 181,000 deaths, predominantly in sub-Saharan Africa [5,6]. *Cryptococcus neoformans* is the most common pathogen in these cases, whereas *C. gattii* infection is seen in immunocompetent populations of the Pacific Northwest in the United States and parts of Australia, thus expanding the profile of at-risk populations, including healthy individuals [7]. In non-endemic regions, however, cryptococcal infections in healthy hosts remain exceedingly rare, often leading to a delayed diagnosis and treatment [8].

Pathophysiology and typical presentation

*Cryptococcus spp.* possess several virulence factors, most notably a thick polysaccharide capsule that aids in immune evasion and melanin production, which protects the fungi against oxidative stress from host immune cells [2]. In immunocompromised hosts, these factors allow progression from a localized pulmonary infection to a disseminated disease, often reaching the CNS and leading to meningoencephalitis [9]. The clinical manifestations include fever, headache, altered mental status, and neurological deficits associated with increased intracranial pressure. Pulmonary cryptococcosis in immunocompromised individuals may present as diffuse interstitial or nodular infiltrates on imaging and can frequently mimic bacterial pneumonia, tuberculosis, or malignancy [10]. In immunocompetent individuals, Cryptococcus infections are generally self-limiting and confined to the lungs, often with asymptomatic or mild symptoms such as cough, fever, and dyspnea. Pulmonary cryptococcosis in these cases may be misinterpreted as bacterial or viral pneumonia, with symptoms often resolving spontaneously [2].

Atypical presentation in immunocompetent hosts

Pulmonary cryptococcosis in immunocompetent hosts remains a diagnostic challenge due to its rarity and nonspecific presentation. Unlike immunocompromised patients, where it typically follows a rapid, severe course, infection in healthy individuals may present with chronic or indolent symptoms that overlap with more common respiratory conditions, leading to misdiagnosis [3]. Increasing recognition of such cases in endemic areas of *C. gattii* infection suggests a potential host-pathogen interaction unique to this species, which may explain its ability to infect healthy hosts [4]. Additionally, genetic polymorphisms influencing immune function have been proposed as a contributing factor in cases where no overt immunosuppression is present, though further research is required to substantiate these findings [5].

## Case presentation

A 39-year-old non-smoking Caucasian male with a BMI of 33.6 was admitted to the emergency department (ED) for worsening shortness of breath, productive cough, fever, fatigue, and loss of appetite. His medical history was notable for obstructive sleep apnea (OSA), with poor compliance with CPAP therapy. Before this admission, he had been diagnosed with pneumonia and treated with a course of amoxicillin and azithromycin. However, his symptoms did not improve, leading to a return visit to the ED 10 days later. He was treated with intravenous antibiotics at that time and subsequently discharged on cefuroxime and doxycycline. The patient reported no chest pain, palpitations, abdominal pain, nausea, vomiting, recent travel, or sick contacts.

**Figure 1:**
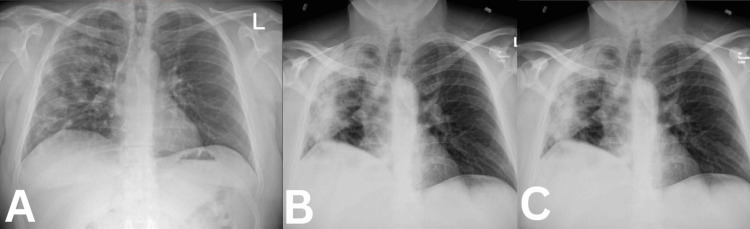
Chest X-rays Chest X-ray demonstrated extensive consolidation in the right lung, suggestive of pneumonia (Figure 1 A) while repeat chest X-rays revealed areas of consolidation in the right lung and apex with associated atelectasis (Figures 1 B, 1C).

On physical examination, the patient was tachycardic, with a heart rate of 120 bpm, and hypoxic, with an oxygen saturation of 90% on room air. Auscultation revealed crackles and diminished breath sounds in the right lung. Laboratory tests revealed leukocytosis, with a white blood cell (WBC) count of 13.69 x 10^9/L. A chest X-ray demonstrated extensive consolidation in the right lung, suggestive of pneumonia (Figure 1A). Given these findings, he was initiated on a combination of piperacillin/tazobactam and doxycycline. During his hospital stay, the patient remained febrile and tachycardic, with persistent cough and shortness of breath upon exertion. His oxygen saturation dropped to the 88% range during ambulation. Laboratory tests showed a normal procalcitonin level, negative viral panels, and negative blood cultures. His WBC count continued to rise, reaching 21 x 10^9/L, with signs of transaminitis. Repeat chest X-rays revealed areas of consolidation in the right lung and apex with associated atelectasis (Figures 1B, 1C).

A follow-up CT scan with intravenous contrast four days after admission showed a similar worsening of multi-lobar pneumonia and a progressive right upper lobe consolidation (Videos 1, 2), prompting a change in antibiotics from piperacillin/tazobactam to meropenem, along with the addition of vancomycin and methylprednisolone due to concerns about inflammatory interstitial pneumonia.

**Video 1:** Contrast enhanced computed tomography chest (transverse section)

**Video 2:** Contrast enhanced computed tomography chest (coronal section)

The patient underwent a comprehensive evaluation, with laboratory findings detailed in Table 1. Given a family history of cystic fibrosis in his maternal aunt, tests for stool elastase, alpha-1 antitrypsin deficiency, and cystic fibrosis transmembrane conductance regulator (CFTR) gene mutations were also conducted. Strikingly, the patient’s cryptococcal antigen screen came back positive, with a titer of 1:320, confirmed with a second titer at 1:160 the following day.

**Table 1:** Lab reports PCR: Polymerase chain reaction, MRSA (PCR): Methicillin-resistant Staphylococcus aureus polymerase chain reaction, CFTR: Cystic fibrosis transmembrane conductance regulator, RSV RNA PCR: Respiratory syncytial virus ribonucleic acid polymerase chain reaction

Laboratory Investigations	Results
Microbiological Investigations	
Sputum Cultures	Negative
MRSA (PCR)	Negative
Legionella Panel	Negative
Streptococcus pneumoniae (PCR)	Negative
Fungitell	Negative
Histoplasma Antigen	Negative
Aspergillus Antibodies	Negative
TB Spot	Negative
Cryptococcal Antigen	Positive
Genetic and Enzymatic Tests	
Stool Elastase	Negative
Alpha-1 Antitrypsin Deficiency	Negative
CFTR Gene Mutations	Negative
Inflammatory Markers	
Erythrocyte Sedimentation Rate (ESR)	63 mm/hr (Normal levels: 0-15 mm/hr)
C-reactive protein (CRP)	51.95 mg/L (Normal levels: <10)
Procalcitonin	<0.10 ng/mL (Normal levels: <0.10 ng/mL)
Viral, Fungal, Bacterial Panels	
Adenovirus DNA	Not Detected
Coronavirus 229E (not COVID-19)	Not Detected
Coronavirus NL63 (not COVID-19)	Not Detected
Coronavirus HKU1 (not COVID-19)	Not Detected
Coronavirus OC43 (not COVID-19)	Not Detected
Human Metapneumovirus	Not Detected
Human Rhinovirus/Enterovirus	Not Detected
Influenza A H3	Not Detected
Influenza A H1	Not Detected
Influenza A H1N1/pdm09	Not Detected
Parainfluenza 1, 2	Not Detected
Parainfluenza 3, 4	Not Detected
Influenza A	Not Detected
Respiratory Syncytial Virus	Not Detected
Bordetella pertussis	Not Detected
Chlamydophila pneumoniae	Not Detected
Mycoplasma pneumoniae	Not Detected
COVID-19 (SARS-CoV-2)	Not Detected
COVID-19/Coronavirus RNA PCR	Not Detected
Influenza A, B RNA PCR	Not Detected
RSV RNA PCR	Not Detected
Chlamydia/Chlamydophila Tests	
Chlamydophila psittaci IgM Ab Titer	≤1:20
Chlamydophila psittaci (IgG)	≤1:64
Chlamydia trachomatis (IgM)	≤1:20
Chlamydia trachomatis (IgG)	≤1:64
Chlamydophila pneumoniae IgM Ab Titer	≤1:20
Chlamydophila pneumoniae IgG Ab Titer	≤1:64
Aspergillus Tests	
Aspergillus Ag EIA	Negative
Aspergillus Galactomannan Index	0.04
Cryptococcal Antigen Tests	
Cryptococcal Titre	1:320
Cryptococcal Titre (Repeat)	1:160
HIV Test	
HIV 1/0/2 Ab/Ag (Screen)	Non-Reactive

Given the non-resolving clinical picture, the decision was made to proceed with bronchoscopy, bronchoalveolar lavage (BAL), and transbronchial biopsies. Histopathological analysis of the transbronchial biopsy demonstrated an organizing pneumonia pattern consistent with secondary infection, with findings compatible with Cryptococcus. At this point, vancomycin was discontinued, while meropenem, doxycycline, and steroids were continued. Bronchial washing analysis revealed occasional yeast forms on BAL silver staining, though mucicarmine staining was negative. The presence of cryptococcal antigen in serum samples, combined with the patient’s indolent but persistent pulmonary infiltrates, strongly suggested a diagnosis of pulmonary cryptococcal infection. Since *Cryptococcus* species are neurotropic, a lumbar puncture was performed to rule out cryptococcal meningitis, which yielded negative results. Following confirmation of pulmonary cryptococcosis, antibiotics were discontinued, and the patient was started on fluconazole at a dosage of 400 mg (6mg/kg) per day orally. Over the following weeks, the patient’s symptoms gradually improved with antifungal therapy, and his pulmonary status stabilized. Steroid therapy was eventually discontinued, and fluconazole treatment was continued as planned for a 12-month course.

## Discussion

This discussion explores in depth the nuances of atypical cryptococcosis presentations, diagnostic challenges, and therapeutic strategies, supplemented by the latest research findings. Cryptococcosis typically occurs in immunocompromised individuals, though it can also present in immunocompetent hosts under specific conditions. *Cryptococcus gattii*, in particular, has shown a higher propensity for infecting immunocompetent hosts, especially in endemic areas [1]. The pathology in immunocompetent hosts typically involves localized pulmonary infections, where immune defenses contain the fungus in granulomas or nodular formations, effectively preventing systemic dissemination. These granulomas, while useful in containing the infection, can be indolent and persist for extended periods, leading to chronic, non-specific respiratory symptoms such as cough and dyspnea, as seen in our patient [2]. This feature contrasts with the rapid dissemination observed in immunocompromised hosts, where immune suppression allows *Cryptococcus* to spread more freely to other organs, particularly the CNS.

The immune response includes the activation of alveolar macrophages and T-cell-mediated immunity. Studies have shown that *C. gattii* may modulate this immune response by altering cytokine production, potentially delaying clearance [3]. Additionally, some immunocompetent hosts exhibit genetic predispositions that may subtly impair their ability to clear *Cryptococcus* infections, such as specific polymorphisms in immune response genes. For example, polymorphisms in genes like toll-like receptor 4 (TLR4) and dectin-1 have been associated with an increased susceptibility to fungal infections, suggesting that subtle immune variations may play a role in certain cases [4]. However, these mechanisms require further research to understand their exact contributions.

Diagnostic challenges: imaging, serology, and histopathology

Diagnosing cryptococcosis in immunocompetent hosts can be particularly challenging. In our case, the initial misdiagnosis as bacterial pneumonia underscores the need for heightened clinical suspicion, especially in cases unresponsive to conventional antibiotics. Imaging, while essential, provides non-specific results. In this patient, CT scans revealed multi-lobar pneumonia and progressive consolidation, which is unique to cryptococcosis but a common finding seen in bacterial infections, malignancies, or other chronic lung diseases [5]. The nodular and mass-like opacities associated with pulmonary cryptococcosis that were somewhat missing here further complicate the diagnosis. By resembling neoplastic lesions, it warrants invasive diagnostic procedures like biopsies.

High-resolution computed tomography (HRCT) helps detect subtle pulmonary nodules or ground-glass opacities, which are often present in fungal infections. A study by Huang et al. showed that patients with pulmonary cryptococcosis often exhibit localized nodular opacities or areas of low attenuation on HRCT, which are sometimes accompanied by halo signs, a finding indicative of fungal infections but not exclusively diagnostic [6].

Cryptococcal antigen serology in cases limited to pulmonary involvement has its limitations. The antigen may not reach detectable levels in serum, especially in early infection stages or when the infection is localized [7]. However, in this case, the patient’s positive cryptococcal antigen titer (1:320) provided critical evidence supporting the diagnosis. A subsequent bronchoalveolar lavage (BAL) and transbronchial biopsy confirmed the infection. Thus, combining imaging with serology and histopathology yields the highest diagnostic accuracy, particularly in immunocompetent patients with localized pulmonary disease [8].

Histopathological analysis from BAL or biopsy samples can confirm the diagnosis even when serological tests are inconclusive. Silver staining, which highlights the polysaccharide capsule of *Cryptococcus*, is a reliable method for identifying the pathogen in tissue samples. The patient’s BAL sample showing occasional yeast forms on silver staining provided definitive evidence of cryptococcal infection, reinforcing the utility of invasive diagnostics when initial non-invasive tests are inconclusive [9].

Implications of persistent symptoms and immune-driven inflammation

In immunocompetent patients, it is frequently associated with chronic inflammation manifesting as prolonged elevated levels of inflammatory markers. In this case, the patient displayed elevated C-reactive protein (CRP) levels and leukocytosis, which persisted despite treatment with antibiotics and antifungals, indicating an atypical or chronic infectious process [10]. Such prolonged inflammation may result in the formation of granulomas, causing residual lung abnormalities that resolve over time [2]. For clinicians, this is indicative of an atypical infectious process rather than bacterial resistance and is critical in differentiating fungal infections in cases unresponsive to standard therapies.

Treatment strategies: efficacy and considerations for extended therapy

Fluconazole, an azole antifungal, is the primary agent for non-CNS pulmonary cryptococcosis in immunocompetent hosts. It acts by inhibiting fungal ergosterol synthesis, thereby disrupting cell wall integrity [3]. The treatment duration varies depending on disease severity; however, extended therapy, often spanning 6-12 months, is recommended to prevent recurrence and ensure complete fungal clearance in cases with significant pulmonary involvement [4]. Studies have shown that fluconazole monotherapy is often sufficient for isolated pulmonary cryptococcosis, providing durable responses with minimal side effects, particularly in immunocompetent hosts [5]. Amphotericin B is typically reserved for disseminated cryptococcal infections due to its potential nephrotoxicity and other side effects. Flucytosine, combined with amphotericin B, is reserved for severe cases with CNS involvement, where rapid fungal clearance is necessary. Immunocompetent patients with limited disease generally do not require such aggressive regimens, as fluconazole alone can achieve adequate fungal control in localized infections [6]. Corticosteroids can be used to manage inflammatory symptoms if a patient exhibits signs of organizing pneumonia. In this case, the patient was initially treated with methylprednisolone due to concerns for inflammatory interstitial pneumonia. While steroids can reduce inflammation, they also suppress the immune response, potentially allowing fungal persistence if not paired with effective antifungal therapy. Hsu et al. suggest that steroids, although occasionally useful, should be discontinued as soon as antifungal treatment begins to take effect, preventing unnecessary immune suppression [7].

Prognosis and long-term management

The prognosis in immunocompetent patients is generally favorable when diagnosed and treated promptly. Long-term follow-up with periodic imaging and inflammatory marker monitoring is recommended to assess treatment response and detect any recurrence [8]. Serial chest CTs can be used to confirm lesion resolution over time, with continued fluconazole therapy until imaging findings indicate full resolution.

For immunocompetent patients with persistent lesions or symptoms, extending fluconazole treatment beyond the standard 6-12 months may be necessary. Studies emphasize that such extended therapy in cases of incomplete radiological or clinical response effectively prevents recurrence and aids in lesion resolution [9]. In areas where *C. gattii* is endemic, clinicians are encouraged to consider extended treatment and follow-up, as recurrence rates may be higher in these cases. Increased clinical awareness of *C. gattii* and its potential to affect immunocompetent individuals, especially in non-endemic regions, may lead to more timely diagnoses and improved outcomes [2].

## Conclusions

This study highlights the importance of identifying atypical presentations of cryptococcosis, especially in immunocompetent individuals, where the condition often mimics more common diseases such as bacterial pneumonia or malignancies. It emphasizes the value of a thorough diagnostic process that combines imaging, serological tests, and histopathological analysis to address the challenges posed by vague clinical and radiological findings. Clinicians are reminded to remain vigilant in cases of persistent or treatment-resistant pulmonary infections, as early recognition and appropriate antifungal therapy are crucial for better patient outcomes. While immunocompetent patients generally have a good prognosis, the study stresses the importance of comprehensive care, including prolonged antifungal treatment, careful monitoring for inflammatory complications, and ongoing follow-up to ensure complete recovery and prevent relapse. The findings underscore the need for a personalized approach to managing this complex fungal infection, taking into account the intricate relationship between the host’s immune response and the characteristics of the pathogen.
